# Case control study of the factor V Leiden and factor II G20210A mutation frequency in women with recurrent pregnancy loss

**Published:** 2013-01

**Authors:** Majid Teremmahi Ardestani, Hossein Hadi Nodushan, Abbas Aflatoonian, Nasrin Ghasemi, Mohammad Hasan Sheikhha

**Affiliations:** 1 *Department of Immunology, Shahid Sadoughi University of Medical Sciences, Yazd, Iran.*; 2 *Department of Obstetrics and Gynecology, Research and Clinical Center for Infertility, Yazd Reproductive Sciences Institute, Shahid Sadoughi University of Medical Sciences, Yazd, Iran.*; 3 *Department of Medical Genetics, Research and Clinical Center for Infertility, Yazd Reproductive Sciences Institute, Shahid Sadoughi University of Medical Sciences, Yazd, Iran. *

**Keywords:** *Factor V Leiden*, *Factor II G20210A Mutation*, *Recurrent Abortion*

## Abstract

**Background: **Recurrent pregnancy loss (RPL) caused by various genetic and non-genetic factors. After chromosome abnormality, thrombophilia is one of the most important genetic factors that could cause RPL. Factor V Leiden and factor II G20210A mutation were the most common mutations cause thrombophilia in the world.

**Objective:** The purpose of this study was to determine the frequency of factor V Leiden and prothrombine gene mutations in women with RPL compared with women who had uneventful pregnancies.

**Materials and Methods:** This case control study evaluates the frequency of factor V-Leiden and factor II G20210 genotypes in 80 women with two or more pregnancy losses, compared with 80 women without adverse pregnancy outcome. The mutations were assessed by PCR-RFLP.

**Results:** Frequency of the factor V Leiden among cases was 2.5%, which was higher than controls (1.25%), but the difference was not significant. No factor II G20210 mutation was found among cases and controls.

**Conclusion:** These data did not confirm that factor V Leiden and factor II G20210 mutation might play a role in recurrent pregnancy loss in Iranian women.

## Introduction

Recurrent pregnancy loss (RPL) has traditionally been defined by two or more consecutive pregnancy losses before 20 weeks gestation. RPL has been estimated to occur in approximately 1% of all couples (1). Recurrent pregnancy loss causes by various genetic and non-genetic factors, which include uterine and cervical anatomic abnormalities, ovarian dysfunction, endocrine problems, immunologic abnormalities, chromosome abnormalities and thrombophilia (2).

Thrombophilia usually causes by point mutation in factor V (G1691A), and factor II prothrombine (G20210A) genes (3). The main underlying mechanism seems to be inhibition of trophoblast differentiation and thrombosis of the maternal side of the placenta (4, 5). The most common inherited thrombophilias include mutation G1691A in factor V gene (FVL) and mutation G20210A in the 3-UTR of gene prothrombine (PRT). Factor V Leiden act by resistance to APC (activated protein C) and PRT G20210A increases plasma prothrombine level (5, 6). 

Prevalence of these mutations varies among different populations and ethnic groups. Factor V Leiden mutation is rare in Asian and African populations and is higher in Eropean populations (5-9% healthy subjects) (7-11). The highest frequency reported in the Eastern Mediterranean region belong of Lebanon (14%) (6). Frequency of the prothrombine mutation varies between 0.7-4% worldwide and affects 3% of healthy subjects in Iranian populations (9).

Previous studies showed a possible association between pregnancy loss and coagulation genetic disorders. Several reports have suggested an increase association between recurrent miscarriage and activated protein C resistance or factor V Leiden (7, 10-13). To verify weather inherited thrombophilia may determine the risk of recurrent abortion; we evaluated the prevalence of FVL and PRT G20210A in a sample of 80 patients with recurrent abortion and in 80 healthy control women.

## Materials and methods

In this case-control study the frequency of factor V Leiden G1691A and prothrombine G20210A mutation were determined in a consecutive series of 80 women referred to Research and Clinical Center for Infertility of Yazd for evaluation of recurrent spontaneous pregnancy loss (case patients). The control group included 80 women with at least two successful pregnancies and no history of pregnancy loss, which matched by age with cases. This case-control study was approved by ethical committee of Research and clinical center for infertility.

Cases were women with unknown RPL, which were chosen after rolling out uterine and cervical anatomical abnormalities, ovarian dysfunction, chromosomal abnormalities, endocrine disorders (diabetes mellitus and hypothyroidism) and immunological problems. Women were excluded if they had any of the mentioned problems.

Peripheral blood was collected into tubes containing EDTA, and genomic DNA was extracted by salting-out method (14). Genomic DNA was amplified by polymerase chain reaction (PCR) using primers previously reported (7, 8). Amplification of factor V gene yields a 267 base pair fragment and PCR product of factor II gene is a 345 base pair fragment. To identify factor V Leiden, PCR product was digested with MNL1 restriction endonucleas enzyme. Wild type allele of factor V gene has two cleavage site for MNLI and after digestion produces 3 fragments (167bp, 63bp, 37bp). While after digestion two fragments are created (200bp, 37bp) in mutant allele.

In factor II gene, PCR product has a length of 345 base pair and it has no cleavage site for Hind III restriction enzyme but in mutant allele 345 bp fragment convert to two fragments (322bp, 23bp) (13).

**Statistical analysis**

Data was processed by SPSS 16.0 Software. Results were compared by x^2 ^test and p-value less than 0.05 assigned statistically significant. Odd ratio and 95% confidence intervals (CI) were calculated.

## Results

In cases 80 women with RPL were evaluated, which their mean age was 28.8 years (range 18-41). In controls mean age was 23.6 years (range 20-36). Differences between ages of cases and controls was not significant (p>0.05). Fifty four women (67.5%) had two consecutive spontaneous abortions and the rest had three or more abortions.

Concerning the factor V Leiden, 2 out of 80 recurrent miscarriage patients and 1 out of 80 controls carried one factor V Leiden mutation (heterozygote). The difference was not significant (p≈0.40). No factor V Leiden homozygote was found in both groups. Concerning the prothrombine G20210A polymorphism, none of the cases and controls carries the prothrombine G20210A mutation (p=1).

**Table I:** Comparison of the prevalence of factor V Leiden and prothrombine G20210A mutation between women with recurrent miscarriage and controls

**Variable**	**Cases (N=80)**	**Controls (N=80)**	**Odd ratio (95%CI)**	p-value*
Factor V Leiden [n (%)]	2 (2.5%)	1 (1.25%)	1 (0.18-22.7)	0.40
Prothrombine [n (%)]	0 (0%)	0 (0%)		1

* Compared by x^2^ test.

**Figure 1:**
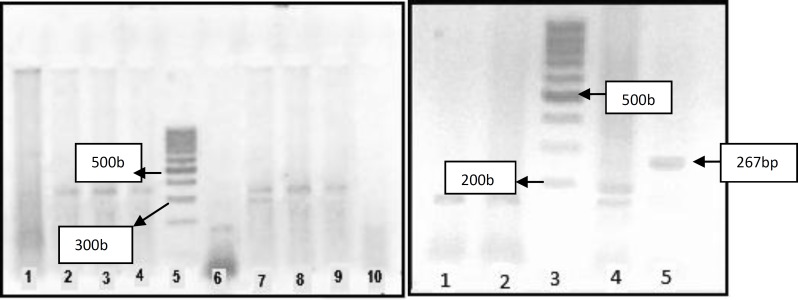
Digestion pattern of PCR products.

Left: (FII, 2, 3, 4, 8, 9: Normal, 7: heterozygous (322, 345bp positive control), 1: No DNA, 5: 100bp DNA ladder.

Right: (FV Leiden, 1, 2: Normal, 4: heterozygous (167, 200bp), 5: uncut sample (267bp).

## Discussion

The results of present study showed factor V Lieden G1691A mutation and prothrombine G20210A mutation were not frequently find in Iranian women with RPL. It is not compatible with the hypothesis that factor V Leiden G1691A and prothrombine G20210A mutations play an important role in pregnancy loss. The description of factor V Leiden as a genetic factor involved in the etiology of thrombosis has stimulated the investigation of this genetic abnormality as a risk factor for recurrent abortion in the world (15, 16). 

In a study was done by Souza *et al* the frequency of factor V Leiden in Brazilian patients was 7.1% vs. 1.6% in controls (17). The frequency of this mutation determined 17% in cases vs. 2% in controls in the United State (18). In contrast, none of the 52 Japanies women with RPL carried factor V Leiden mutation (19).

The frequency of factor V Leiden G1691A in Iranian populations was reported 5.5%, but present data showed lower rate. The frequency of factor II G20210A mutation in Iranian population was 3.1%. However, in present study no factor II mutation in cases and controls was found. Behjati *et al* showed that the frequency of factor V Leiden mutation in iranian patients with infertility and recurrent spontaneous abortion is 30.6% and 20% respctivley. The frequency of the factor II mutation in patients with infertility was 2.8% in RSA it was 4.6% and in control group the rate was 3.2%, the results are disagreed with the present study (20). The frequency of factor V Leiden in Turkey was high (8-10%), while in Kuwait this rate was low (21, 22). The frequency of these two mutation decreases from north to south of Iran, therefore it might explain low frequency of these mutations in present study. 

Further studies should work on other point mutation in these two factors, which might show high frequency in center and north of Iran. Treatment of women with unknown RPL with aspirin and/or heparin showed high rate of the successful pregnancy. It shows that thrombophilia could cause RPL in most of unknown cases.
